# Increased fasting plasma succinate levels are associated with higher resting heart rate in young sedentary adults

**DOI:** 10.3389/fphys.2026.1860592

**Published:** 2026-08-03

**Authors:** Francisco J. Osuna-Prieto, Juan M. A. Alcantara, Abel Plaza-Florido, Joan Vendrell, Sonia Fernández-Veledo, Jonatan R. Ruiz

**Affiliations:** 1 Hospital Universitari Joan XXIII de Tarragona, Institut de Recerca Biomèdica Catalunya Sud (IRB CatSud), Tarragona, Spain; 2 CIBER de Diabetes y Enfermedades Metabólicas Asociadas (CIBERDEM)-Instituto de Salud Carlos III (ISCIII), Madrid, Spain; 3 Department of Education, Faculty of Education Sciences, University of Almería, Almería, Spain; 4 SPORT Research Group (CTS-1024), CIBIS (Centro de Investigación para el Bienestar y la Inclusión Social) Research Center, University of Almería, Almería, Spain; 5 Centro de Investigación Biomédica en Red Fisiopatología de la Obesidad y Nutrición (CIBERobn), Instituto de Salud Carlos III, Madrid, Spain; 6 Research Center for Exercise Medicine and Sleep/Pediatric Exercise and Genomics Research Center, Department of Pediatrics, School of Medicine, University of California Irvine, Irvine, CA, United States; 7 Universitat Rovira i Virgili (URV), Reus, Spain; 8 Department of Physical Education and Sports, Faculty of Sports Science, Sport and Health University Research Institute (iMUDS), University of Granada, Granada, Spain; 9 Instituto de Investigación Biosanitaria (Ibs), Granada, Spain

**Keywords:** autonomic nervous system, cardiometabolic health, heart rate variability, metabolic–autonomic crosstalk, signaling metabolites

## Abstract

**Purpose:**

Succinate is a circulating metabolic intermediate and signaling metabolite associated with several physiological and immunometabolic processes. However, whether circulating succinate relates to resting cardiovascular physiology in humans remains poorly understood. Therefore, we examined the association between fasting plasma succinate concentrations and resting heart rate (HR) and heart rate variability (HRV) parameters in young sedentary adults.

**Methods:**

We analyzed 85 sedentary young adults (18–25 years; 56 women) from the ACTIBATE study cohort (NCT02365129). Plasma succinate was measured enzymatically under fasting conditions. Resting heart rate (HR) and heart rate variability (HRV), non-invasive markers of autonomic function, were measured in fasting, thermoneutral conditions using the Polar RS800CX system.

**Results:**

Higher fasting succinate concentrations showed a modest positive association with resting HR (β=0.213; R²=0.074; P = 0.046), independent of sex and BMI. In exploratory sex-stratified analyses, the association remained significant in men (β=0.379; R²=0.200; P = 0.034) but not in women (β=0.112; R² =–0.024; P = 0.417), although no significant Succinate × Sex interaction was detected. Participants with succinate levels above the cohort median showed a higher resting HR (+4 bpm; 95% CI: +0.1 to +8.3; P = 0.045) compared with those below the median, despite comparable body composition and cardiometabolic profiles (all P≥0.105). There was no association between levels of succinate and any HRV parameters (all P≥0.226).

**Conclusion:**

Elevated fasting plasma succinate is associated with higher resting HR in young sedentary adults. While causality cannot be inferred, these findings suggest that circulating succinate may represent a systemic metabolic marker associated with resting cardiovascular physiology in young adults. Longitudinal and mechanistic studies are warranted to clarify the physiological relevance and directionality of these associations.

## 1 Introduction

Cardiovascular diseases (CVD) remain the leading cause of global mortality despite major advances in prevention and treatment, underscoring the need for early and accessible biomarkers capable of capturing cardiovascular risk before clinical onset (Hoogeveen et al., 2020; Di Cesare et al., 2024). Among emerging candidates, succinate has gained attention as a metabolic intermediate and signaling metabolite involved in intercelullar communication, energy sensing, inflammation, hypoxia responses, and tissue remodeling (Tretter et al., 2016; Keiran et al., 2019; Fernández-Veledo et al., 2021; Villanueva-Carmona et al., 2023; Sabadell-Basallote et al., 2024). We previously reported that elevated fasting plasma succinate associates with classical CVD risk factors, visceral adiposity, and systemic inflammation in young sedentary adults (Osuna-Prieto et al., 2021a), supporting its candidacy as an early biomarker of cardiometabolic status. Importantly, circulating succinate likely reflects mixed contributions from host metabolism, tissue release, and gut microbiota-derived production, and the relative contribution of these sources may vary according to the metabolic and clinical context. While gut microbiota-derived succinate has been particularly linked to obesity-related metabolic dysfunction (Serena et al., 2018), previous analyses involving young sedentary adults did not identify strong associations between circulating succinate and gut microbiota composition (Osuna-Prieto et al., 2021b), suggesting that circulating succinate in this population may reflect broader systemic metabolic processes.

Despite this complexity, accumulating experimental evidence suggests that succinate-related pathways may participate in cardiovascular and inflammatory regulation, including vascular inflammation, oxidative stress, renal signaling, and mitochondrial stress responses (Chouchani et al., 2014; Khamaysi et al., 2019; Xu et al., 2022a; Yuan et al., 2024; Zhang et al., 2025). However, whether circulating succinate is associated with physiological markers of cardiovascular regulation in humans remains unclear.

Building upon these pathophysiological connections, it becomes relevant to explore whether circulating succinate also relates to physiological markers in cardiovascular regulation that precede the clinical onset of disease. Autonomic balance is a clinically relevant marker that can be non-invasively assessed through cardiac measures reflecting sympathetic–parasympathetic activity (Shaffer and Ginsberg, 2017; Olshansky et al., 2023). Among these, resting heart rate (HR) provides a robust and easily measurable index of cardiac autonomic tone, whereas heart rate variability (HRV), defined as beat-to-beat fluctuations in successive cycles, captures the dynamic modulation of autonomic inputs (Shaffer and Ginsberg, 2017; Olshansky et al., 2023). Alterations in autonomic cardiovascular regulation, characterized by elevated resting HR and altered HRV metrics, have been associated with cardiovascular risk in multiple clinical and epidemiological contexts, even in apparently healthy populations (Hillebrand et al., 2013; Tadic et al., 2018). Interestingly, both HR and HRV are linked to inflammatory and neurohumoral pathways that have been associated with succinate-related signaling in experimental models (Pavlov et al., 2003; Ariza et al., 2012). Preclinical evidence suggests that succinate signaling may interact with inflammatory and neuroimmune pathways involved in cardiovascular regulation (Keiran et al., 2019; Krzak et al., 2021). Yet, whether circulating succinate is associated with autonomic cardiovascular regulation in humans remains unknown.

Based on these converging lines of evidence, we hypothesized that higher fasting plasma succinate concentrations are associated with elevated resting HR and reduced HRV in young, sedentary adults. Establishing such a link could provide novel insight into the relationship between circulating metabolic signals and cardiovascular regulation in humans.

## 2 Methods

### 2.1 Study participants

Analyses used baseline data from the ACTIBATE trial (ClinicalTrials.gov ID: NCT02365129) (Sanchez-delgado et al., 2015), which was designed to evaluate the effects of concurrent exercise training on brown adipose tissue (BAT) volume, activity, and radiodensity. In this cross-sectional study, we selected baseline data from 85 young adults (18–25 years old; 56 women) with valid data for HR and HRV parameters. Participants were eligible if they were sedentary (defined as performing less than 20 minutes of moderate-to-vigorous physical activity on fewer than three days per week), non-smokers, had maintained stable body weight for at least three months, and were not taking any medications. Exclusion criteria included diagnosed diabetes, hypertension, or other significant medical conditions that might interfere with or be exacerbated by exercise, pregnancy, use of drugs affecting energy metabolism, or regular exposure to cold environments. This study was approved by the Research Ethics Committee at the University of Granada (approval no. 924) and the *Servicio Andaluz de Salud*. All procedures were conducted in accordance with the Declaration of Helsinki (2013 revision). Oral and written informed consent was obtained from all subjects.

### 2.2 Anthropometry and body composition

Weight and height were measured with participants barefoot and wearing light clothing using a Seca scale and stadiometer (model 799; Electronic Column Scale, Seca, Hamburg, Germany). These measurements were used to calculate body mass index (BMI; kg/m²). Waist circumference (WC) was measured at the narrowest point of the abdomen at the end of a normal exhalation, with participants’ arms relaxed at their sides. If the minimum perimeter could not be identified, the measurement was taken just above the umbilicus in a horizontal plane. WC was measured twice using a plastic measuring tape, and the average of these two measurements was used for analysis. Lean mass, fat mass, and visceral adipose tissue (VAT) mass were assessed using dual-energy X-ray absorptiometry (DXA) with a Discovery Wi scanner (Hologic Inc., Bedford, MA, USA). The fat mass percentage was also derived from the DXA scan.

### 2.3 Blood samples, plasma succinate, and cardiometabolic risk factors

Blood samples were collected from the antecubital vein between 8:00 and 9:00 A.M. following an overnight fast of more than 10 h, under resting conditions. Samples were drawn into Vacutainer^®^ tubes and immediately centrifuged. Serum aliquots were obtained using Vacutainer^®^ SST™ II Advance tubes, while plasma aliquots were collected using Vacutainer^®^ Hemogard™ tubes containing potassium ethylenediaminetetraacetic acid (EDTA) as anticoagulant. All aliquots were stored at −80 °C until analysis. Serum samples were used for cardiometabolic risk factor assessment, whereas plasma samples were used to quantify succinate.

Plasma succinate levels were determined using a specific kit (EnzyChromTM Succinate Assay Kit; BioAssay Systems, Hayward, CA) (Osuna-Prieto et al., 2021a). Glucose concentrations were measured with an AU5832 biochemical analyzer (Beckman Coulter, Brea, CA) using the Beckman Coulter reagent #OSR6521. Insulin levels were determined on a DXI analyzer (Beckman Coulter) with a chemiluminescent reagent (#33410). These measurements were used to calculate the homeostatic model assessment of insulin resistance (HOMA-IR) index (Bonora et al., 2000). Serum total cholesterol, triglycerides, and high-density lipoprotein cholesterol (HDL-C) were quantified using the AU5832 analyzer with reagents #OSR6116, OSR60118, and OSR6187, respectively. Low-density lipoprotein cholesterol (LDL-C) was subsequently estimated using the Friedewald formula: LDL-C = total cholesterol − HDL-C − 0.45 × triglycerides (Kannan et al., 2014). C-reactive protein levels were also measured with the AU5832 analyzer using reagent #OSR6299. Systolic and diastolic blood pressure were measured using an automatic sphygmomanometer (Omron M2; Omron Healthcare, Kyoto, Japan). Blood pressure measurements were taken on three separate days, and the mean values were calculated.

### 2.4 Heart rate and heart rate variability

Subjects arrived at 08:00–09:00 AM to the research center. Then, the heart signal was assessed during 15 min while the participant rested, lying in the supine position on a bed. A Polar RS800CX (Polar Electro, Kempele, Finland) with a sample frequency of 1000 Hz was used to register. Subsequently, the HR signal was processed using the Kubios HRV software (v.3.0.0 [free version], University of Eastern Finland) (Tarvainen et al., 2014), and the medium Kubios filter was applied (Alcantara et al., 2020). Next, we derived the HR, and the following HRV parameters as follows: the square root of the mean of the sum of the squares of successive normal R-R interval differences (RMSSD), the standard deviation of all normal R-R intervals (SDNN), the percentage of pairs of adjacent normal R-R intervals differing by more than 50 milliseconds in the entire recording (pNN50), the absolute power of the low and the high frequency bands (LF and HF respectively), the LF to HF ratio (LF/HF), and the sympathetic:parasympathetic (S:PS) ratio (Plaza-Florido et al., 2022). The latter was calculated as (1000/SD2)/SD1, where SD2 and SD1 are the standard deviation along the longitudinal axis and the transversal axis of the Poincaré plot, respectively (Plaza-Florido et al., 2022). Interpretation of LF/HF-derived indices as direct markers of sympathovagal balance remains controversial and should therefore be interpreted cautiously.

### 2.5 Statistical analysis

The normality of variables was assessed using the Kolmogorov-Smirnov test, alongside visual inspection of histograms and Q-Q plots. As HRV parameters did not follow a Gaussian distribution, they were transformed using the natural logarithm. Between-group differences in anthropometric parameters and cardiometabolic profile (men vs. women; low- vs. high-succinate groups) were assessed using Student’s t-test for independent samples.

We examined the associations between plasma succinate levels (independent variable) and HR and HRV parameters (dependent variables) using multiple linear regression models. To assess potential sex-based interactions, we included Succinate × Sex interaction terms in the models for HR and each HRV parameter. No significant interactions were observed (all P≥0.1); however, sex emerged as a significant independent predictor in several models and was therefore included as a covariate in all subsequent linear regressions and ANCOVAs. BMI, fat mass index (FMI), and visceral adipose tissue (VAT) were also included as covariates, as succinate levels are increased in obesity (Serena et al., 2018) and linked to VAT depots (Osuna-Prieto et al., 2021).

Linear regression analyses assessed associations between plasma succinate and HR, RMSSD, SDNN, pNN50, HF, LF, the LF/HF ratio, and the S:PS ratio, both across the full cohort and after stratifying by sex. Scatter plots depict fitted linear regressions with 95% confidence intervals. Plasma succinate levels were also dichotomized into low and high groups based on the median value, and between-group differences in HR and HRV parameters were examined using analysis of covariance (ANCOVA), adjusting for sex and BMI. For each model, results are reported as standardized β coefficients, adjusted R² values, and P values. All statistical analyses were performed using SPSS (v. 22.0, IBM Corporation, Chicago, IL, USA) and GraphPad Prism (v. 9.5, GraphPad Software, San Diego, CA, USA). Statistical significance was set at P ≤ 0.05.

## 3 Results

**Figure 1:**
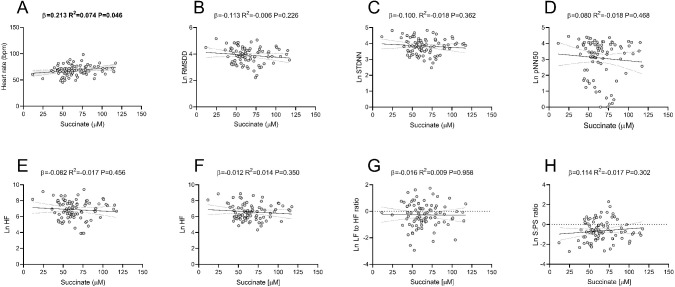
Associations between plasma succinate levels and heart rate and heart rate variability parameters in young, sedentary individuals (n=85). Results are derived from multiple linear regression models adjusted for sex and BMI. Upper plot legends display the standardized regression coefficient (β), the adjusted R²adj, and the P-value. 95% confidence intervals are represented as dashed lines above and below the linear regression lines. Coloured shaded depicts significant associations (P<0.05). **(A)** heart rate; **(B)** root mean square of successive differences (RMSSD); **(C)** standard deviation of NN intervals (SDNN); **(D)** percentage of successive NN intervals differing by more than 50 ms (pNN50); **(E)** high-frequency power (HF); **(F)** low-frequency power (LF); **(G)** low-to-high frequency ratio (LF/HF); and **(H)** inverse ratio of SD1 to SD2. BMI, body mass index.

The characteristics of the study participants are summarized in **Table 1**. In multiple linear regression models adjusted for sex and BMI, fasting plasma succinate concentrations were positively associated with resting HR (β=0.213, R²=0.074, P = 0.046; **Figure 1A**). This association remained significant when further adjusted for FMI or VAT mass (P = 0.036 and P = 0.047, respectively; **Supplementary Table 1**). When analyses were stratified by sex and adjusted for BMI, the association between succinate and resting HR reached nominal statistical significance in men (β=0.379; R²=0.200; P = 0.034), but not in women (β=0.112; R²=–0.024; P = 0.417). However, no significant Succinate × Sex interaction was detected in the overall regression model. Given the unequal sex distribution and limited sample size, these exploratory subgroup analyses should be interpreted cautiously and are presented for descriptive purposes only (**Supplementary Figure S1**) (**Supplementary Table 1**). In contrast, fasting plasma succinate showed no associations with any HRV parameters (all P≥0.226; **Figures 1B–H**) (**Supplementary Table 2**), and additional adjustments for FMI or VAT did not alter these null findings (all P≥0.196; **Supplementary Table 1**).

**Table 1:** Characteristics of the study’s participants (n=85).

	All (11.6-117.3 µM)		Men (n=29) (24.6-117.3 µM)		Women (n=56) (11.6-115.0 µM)		P sex
	Mean	SD	Mean	SD	Mean	SD	
Age (years)	22.1	2.1	22.3	2.4	22.0	2.0	0.528
Sex (n.%)							0.449
Male	29 (34)						
Female	56 (66)						
Body composition							
BMI (kg/m^2^)	25.0	4.6	24.6	4.3	25.2	4.7	0.558
Lean mass index (kg/m^2^)	14.6	2.4	14.8	2.7	14.6	2.3	0.703
Fat mass index (kg/m^2^)	9.0	2.9	8.4	2.6	9.3	3.0	0.165
Fat mass (%)	36.1	7.0	34.5	7.0	37.0	7.0	0.129
VAT mass (g)	356.9	191.2	355.3	187.5	357.7	194.8	0.957
Cardiometabolic risk factors							
Glucose (mg/dL)	87.7	6.5	86.9	6.2	88.1	6.7	0.443
Insulin (µIU/mL)	8.3	4.8	7.5	3.4	8.8	5.3	0.239
HOMA-IR	1.8	1.2	1.6	0.8	2.0	1.4	0.237
Total cholesterol (mg/dL)	164.7	31.8	166.0	29.6	164.1	33.1	0.795
HDL-C (mg/dL)	52.6	12.3	51.8	11.1	53.0	13.0	0.664
LDL-C (mg/dL)	95.2	26.7	98.4	26.3	93.6	26.9	0.436
Triglycerides (mg/dL)	89.0	55.3	79.9	36.5	93.5	62.5	0.289
APOA1 (mg/dL)	146.0	31.9	141.9	24.5	148.3	35.4	0.409
APOB (mg/dL)	69.1	21.5	75.1	23.6	65.7	19.8	0.068
GPT (IU/L)	19.2	19.5	22.5	31.0	17.5	9.6	0.266
GGT (IU/L)	19.8	21.9	24.4	34.3	17.6	11.4	0.180
ALP (IU/L)	73.2	20.5	70.7	15.2	74.4	22.7	0.430
Creatinine (mg/dL)	0.8	0.1	0.7	0.1	0.8	0.1	0.087
Creatine kinase (μmol/L)	98.0	66.1	99.1	90.8	97.4	50.4	0.912
C-reactive protein (mg/L)	2.8	3.7	2.2	2.1	3.1	4.3	0.335
Systolic blood pressure (mmHg)	116.2	11.0	124.3	10.3	112.1	9.0	**<0.001**
Diastolic blood pressure (mmHg)	70.8	7.2	71.7	9.9	70.3	5.5	0.418

Data presented as mean and standard deviation (SD). ALP, alkaline phosphatase; APOA1, apolipoprotein A1; APOB, apolipoprotein B; BMI, body mass index; GGT, gamma-glutamyl transferase; GPT, glutamic pyruvic transaminase; HDL-C, high-density lipoprotein cholesterol; HOMA-IR, homeostatic model assessment of insulin resistance index; LDL-C, low-density lipoprotein cholesterol; VAT, visceral adipose tissue.Bold values indicate statistically significant differences (*P* < 0.05).

**Figure 2:**
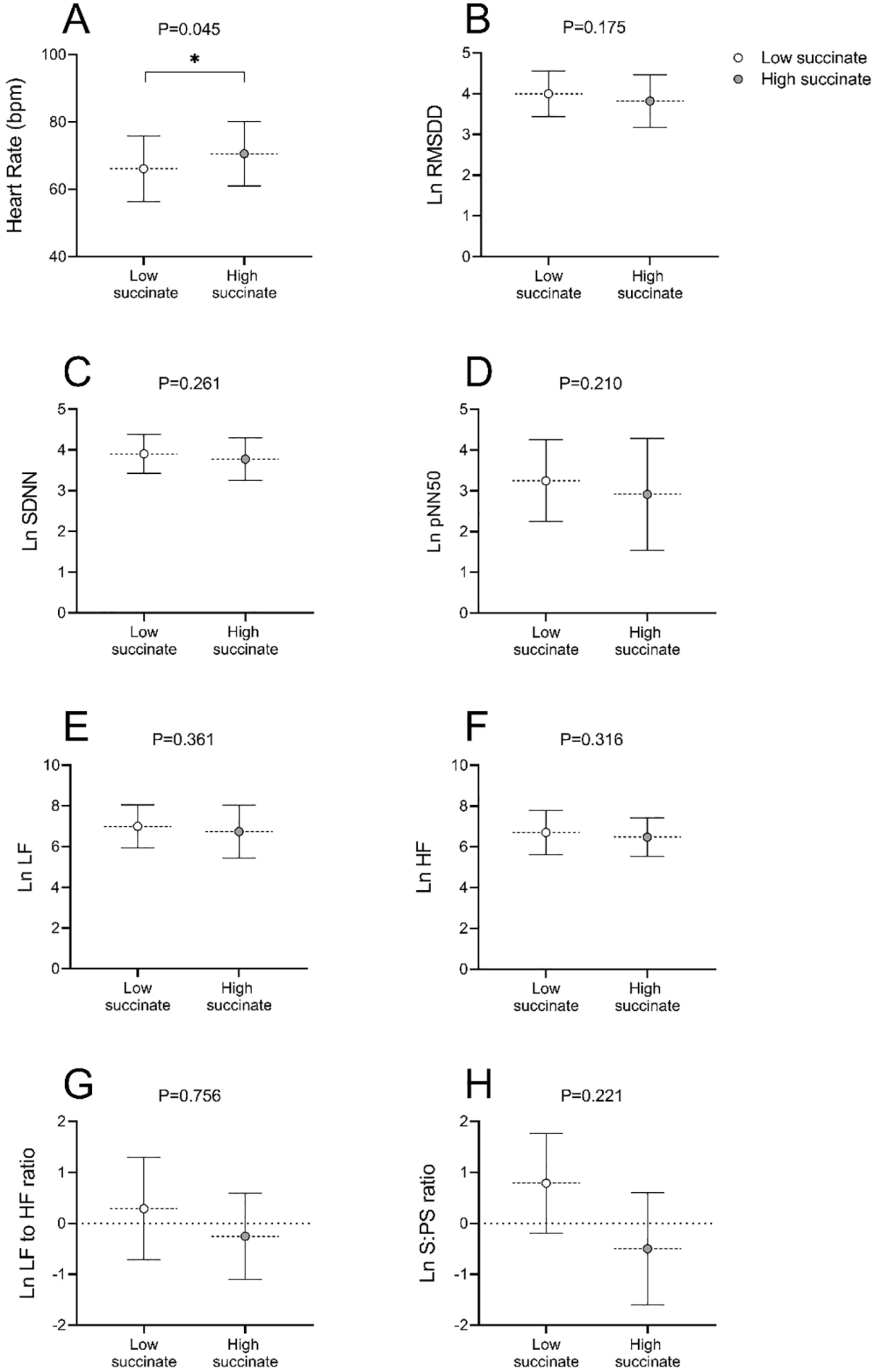
Comparison of heart rate (HR) and heart rate variability parameters between individuals with low (n=43) and high (n=42) plasma succinate levels, dichotomized by the median succinate concentration in young, sedentary individuals. Results are presented as adjusted means with 95% confidence intervals and P values from ANCOVA models including sex and BMI as covariates. Panels show: **(A)** heart rate; **(B)** root mean square of successive differences; **(C)** standard deviation of NN intervals; **(D)** percentage of successive NN intervals differing by more than 50 ms; **(E)** high-frequency power; **(F)** low-frequency power; **(G)** sympathetic to parasympathetic ratio; and **(H)** inverse ratio of SD1 to SD2. These are established parameters of heart rate variability (HRV), including RMSSD, SDNN, pNN50, HF, LF, and LF/HF.

To further evaluate the physiological relevance of this association, participants were divided into two groups according to the cohort median plasma succinate concentration (61.5 µM). Succinate levels ranged from 11.6 to 61.3 µM in the low-succinate group and from 61.7 to 117.3 µM in the high-succinate group. As shown in **Supplementary Table 1**, no significant differences were detected between groups in body composition or conventional CVD risk markers. However, after adjustment for sex and BMI, the high-succinate group exhibited a significantly higher resting HR than the low-succinate group (+4 bpm; 95% CI: +0.1 to +8.3 bpm; P = 0.045; **Figure 2A**). In contrast, none of the HRV parameters differed significantly between groups (**Figures 2B–H**; all P≥0.176).

## 4 Discussion

To the best of our knowledge, this is the first study to examine the association between fasting plasma succinate concentrations and resting HR and HRV parameters in young sedentary adults. We observed a modest positive association between circulating succinate levels and resting HR, whereas no significant associations were detected with HRV metrics. In exploratory analyses, the association with resting HR appeared stronger in men, although no significant Succinate × Sex interaction was observed. Consistently, participants with plasma succinate concentrations above the cohort median exhibited a modest but significantly higher resting HR compared with those below the median, despite comparable body composition and cardiometabolic profiles. Collectively, these findings suggest that circulating succinate may reflect systemic metabolic states associated with resting cardiovascular physiology in young adults.

The potential physiological relevance of this observation should be interpreted in the context of epidemiological studies linking resting HR with long-term cardiovascular outcomes. Even small elevations in resting HR predict long-term cardiovascular risk: a 5-bpm increase associates with a 17% higher risk of cardiovascular mortality (Hozawa et al., 2004), while a 10-bpm increment corresponds to approximately 15% higher cardiovascular morbidity and 17% higher all-cause mortality (Aune et al., 2017). Importantly, this association persisted after adjusting for both FMI and VAT, adiposity markers previously shown to influence circulating succinate levels (Serena et al., 2018; Osuna-Prieto et al., 2021a). Although mechanistic interpretation is beyond the scope of the present study, preclinical evidence has suggested several pathways through which succinate-related signaling may influence cardiovascular physiology. In experimental models, activation of SUCNR1 by succinate in the kidney has been reported to promote renin release, potentially contributing to vascular and cardiovascular responses (Sadagopan et al., 2007; Ariza et al., 2012). At the vascular level, succinate-related pathways have also been implicated in inflammatory and vascular responses (Xu et al., 2022b) and in cardiovascular and inflammatory processes in preclinical models, although whether these mechanisms operate in humans under physiological conditions remains unclear (Milliken et al., 2022). While not directly assessed here, these experimental findings may provide a potential biological context for interpreting the observed association.

In exploratory sex-stratified analyses, the association between plasma succinate and resting HR reached nominal statistical significance in men but not in women. However, no significant Succinate × Sex interaction was detected, and the unequal sex distribution, particularly the smaller number of men, limits the interpretation of these subgroup findings. Therefore, these observations should be considered preliminary and hypothesis-generating, requiring confirmation in larger and more balanced cohorts. Although sex-related differences in autonomic, inflammatory, and cardiovascular regulation have been previously described (Benetos et al., 1999; Dart et al., 2002; Williams et al., 2019), the present study was not powered to formally assess sex-specific associations, and no firm conclusions regarding sex-dependent effects can be drawn.

The lack of association between plasma succinate and HRV may be compatible with the young and apparently healthy profile of the cohort. HRV alterations typically emerge later in life and reflect more pronounced alterations in autonomic cardiovascular regulation (Umetani et al., 1998), whereas resting HR may capture subtler, earlier shifts in autonomic regulation during young adulthood. Supporting this interpretation, a previous study in young adults reported that resting HR, but not HRV, was inversely associated with cardiorespiratory fitness (Plaza-florido et al., 2021), a well-established predictor of CVD risk and all-cause mortality (Fox et al., 2007; Cooney et al., 2010; Ross et al., 2016; Zhang et al., 2016). In contrast, HRV appears to gain prognostic value in middle-aged and older populations, where reduced vagal modulation and sympathetic predominance contribute to cardiometabolic disease progression (Tsuji et al., 1996; Dekker et al., 2000). Thus, in young healthy adults, resting HR may represent a more detectable physiological correlate than HRV metrics under the present experimental conditions, potentially reflecting systemic metabolic states associated with cardiovascular physiology. Nevertheless, while preclinical evidence implicates succinate in cardiovascular control via renal, vascular, and cardiac pathways, whether these mechanisms are operative in humans remains to be elucidated. Importantly, the observed associations were modest in magnitude and should therefore be interpreted cautiously.

## 5 Limitations

First, plasma succinate was measured as total circulating succinate; therefore, we cannot determine the relative contribution of mitochondrial metabolism, host tissue release, or gut microbiota-derived production to circulating levels. Although previous analyses in a closely related ACTIBATE cohort (Osuna-Prieto et al., 2021b) did not identify strong associations between circulating succinate and gut microbiota composition in young sedentary adults, microbiome data were not available in the present study and therefore microbiota contributions cannot be excluded. Second, the cross-sectional design precludes causal inference; thus, longitudinal studies are required to determine whether circulating succinate predicts subsequent autonomic or cardiovascular alterations. Third, the relatively modest sample size limits statistical power, generalizability, and the robustness of exploratory sex-stratified analyses, and the possibility of type I error cannot be excluded. Fourth, the cohort consisted exclusively of young sedentary adults; therefore, the findings cannot be extrapolated to older or physically active individuals, or to populations with established cardiovascular disease. In addition, because plasma succinate was measured at a single fasting time point, temporal variability and acute physiological influences on circulating concentrations could not be evaluated. Finally, dietary intake was not recorded, and therefore potential dietary influences on fasting plasma succinate concentrations cannot be excluded.

## 6 Conclusion

In young sedentary adults, fasting plasma succinate levels are positively associated with resting HR, but not with heart rate variability. While causality cannot be inferred, these findings suggest that circulating succinate may reflect systemic metabolic states associated with resting cardiovascular physiology in young adults. Longitudinal and mechanistic studies are warranted to confirm these associations and clarify their clinical significance.

## Data Availability

The original contributions presented in the study are included in the article/**Supplementary Material**. Further inquiries can be directed to the corresponding authors.
