# Supplementary material for: Increased fasting plasma succinate levels are associated with higher resting heart rate in young sedentary adults
Source: Front Physiol. 2026 Aug 3;17:1860592. doi: 10.3389/fphys.2026.1860592 (PMC13477974; doi:10.3389/fphys.2026.1860592)
Supplement: Supplementary file 1 [file DataSheet1.docx]

**Table S1.** Associations between plasma succinate levels and heart rate and heart rate variability parameters in young individuals stratified by sex (n=85).

|  | Model 1: adjusted by BMI | | | | | Model 2: adjusted by FMI | | | | | Model 3: adjusted by VAT | | | | | |
| --- | --- | --- | --- | --- | --- | --- | --- | --- | --- | --- | --- | --- | --- | --- | --- | --- |
|  | Standardized β | R | R^2^ | Adjusted R^2^ | P | Standardized β | R | R^2^ | Adjusted R^2^ | P value | | Standardized β | R | R^2^ | Adjusted R^2^ | P value |
| Hear rate | 0.213 | 0.322 | 0.104 | 0.071 | 0.046 | 0.224 | 0.355 | 0.112 | 0.080 | 0.036 | | 0.213 | 0.310 | 0.096 | 0.062 | 0.047 |
| Ln RMSDD | -0.133 | 0.153 | 0.023 | -0.013 | 0.228 | -0.143 | 0.172 | 0.030 | -0.006 | 0.196 | | -0.133 | 0.139 | 0.019 | -0.017 | 0.232 |
| Ln STDNN | -0.100 | 0.136 | 0.018 | -0.018 | 0.365 | -0.102 | 0.134 | 0.018 | -0.018 | 0.361 | | -0.098 | 0.173 | 0.030 | -0.006 | 0.373 |
| Ln p NN50 | -0.080 | 0.131 | 0.017 | -0.019 | 0.468 | -0.090 | 0.133 | 0.018 | -0.019 | 0.419 | | -0.080 | 0.084 | 0.007 | -0.030 | 0.472 |
| Ln LF | -0.102 | 0.218 | 0.048 | 0.12 | 0.251 | -0.095 | 0.207 | 0.043 | 0.007 | 0.386 | | -0.100 | 0.224 | 0.050 | 0.015 | 0.361 |
| Ln HF | -0.083 | 0.123 | 0.015 | -0.021 | 0.457 | -0.093 | 0.146 | 0.021 | -0.015 | 0.400 | | -0.082 | 0.095 | -0.28 | -0.082 | 0.463 |
| Ln LF to HF ratio | -.006 | 0.291 | 0.085 | 0.051 | 0.957 | 0.015 | 0.295 | 0.087 | 0.053 | 0.885 | | -0.005 | 0.191 | 0.036 | 0.001 | 0.967 |
| Ln S:PS ratio | 0.114 | 0.131 | 0.017 | -0.019 | 0.305 | 0.118 | 0.139 | 0.019 | -0.017 | 0.288 | | 0.112 | 0.153 | 0.023 | -0.013 | 0.310 |

Results are derived from multiple linear regression models adjusted for BMI (model 1), FMI (model 2), and VAT (model 3). **Abbreviations:** BMI, body mass index; FMI, fat mass index; HF, high-frequency power; LF, low-frequency power; LF/HF ratio, ratio of low- to high-frequency power; pNN50, percentage of successive NN intervals differing by more than 50 ms; RMSSD, root mean square of successive differences; SDNN, standard deviation of NN intervals; S:PS ratio, sympathetic-to-parasympathetic ratio (inverse ratio of SD1 to SD2); VAT, visceral adipose tissue.

**Table S2:** Characteristics of the study participants stratified by circulating succinate levels, categorized into a low succinate group (n=43) and a high succinate group (n=42).

|  | Low succinate  (11.6-61.3 µM) | | High succinate  (61.7-117.3 µM) | | P  group |
| --- | --- | --- | --- | --- | --- |
|  | Mean | SD | Mean | SD |  |
| Age (years) | 22.1 | 2.3 | 22.2 | 2.0 | 0.812 |
| Sex (male/female) |  | |  | | 0.543 |
| *Male* | 16 (37.2) | | 13 (31) | |  |
| *Female* | 27 (62.8) | | 29 (69) | |  |
| Body composition |  |  |  |  |  |
| BMI (kg/m2) | 25.0 | 4.9 | 24.9 | 4.2 | 0.939 |
| Lean mass index (kg/m2) | 14.6 | 2.6 | 14.7 | 2.3 | 0.868 |
| Fat mass index (kg/m2) | 9.1 | 3.2 | 8.8 | 2.6 | 0.698 |
| Fat mass (%) | 36.4 | 7.7 | 35.9 | 6.3 | 0.759 |
| VAT mass (g) | 366.8 | 209.2 | 346.8 | 172.9 | 0.633 |
| Cardiometabolic risk factors |  |  |  |  |  |
| Glucose (mg/dL) | 88.0 | 6.5 | 87.3 | 6.5 | 0.656 |
| Insulin (µIU/mL) | 8.8 | 5.6 | 7.8 | 3.8 | 0.353 |
| HOMA-IR | 2.0 | 1.4 | 1.7 | 1.0 | 0.368 |
| Total cholesterol (mg/dL) | 169.1 | 31.8 | 160.1 | 31.5 | 0.200 |
| HDL-C (mg/dL) | 52.1 | 12.3 | 53.1 | 12.4 | 0.724 |
| LDL-C (mg/dL) | 99.1 | 28.2 | 91.0 | 24.6 | 0.168 |
| Triglycerides (mg/dL) | 97.2 | 65.5 | 80.3 | 41.1 | 0.164 |
| APOA1 (mg/dL) | 143.1 | 29.7 | 148.8 | 34.1 | 0.444 |
| APOB (mg/dL) | 71.9 | 21.0 | 66.3 | 22.0 | 0.270 |
| GPT (IU/L) | 17.4 | 9.5 | 21.0 | 26.2 | 0.406 |
| GGT (IU/L) | 19.4 | 13.3 | 20.3 | 28.5 | 0.848 |
| ALP (IU/L) | 72.6 | 18.8 | 73.8 | 22.3 | 0.790 |
| Creatinine (mg/dL) | 0.8 | 0.2 | 0.8 | 0.1 | 0.715 |
| Creatine kinase (μmol/L) | 92.8 | 54.1 | 103.3 | 77.0 | 0.470 |
| C-reactive protein (mg/L) | 3.0 | 4.5 | 2.5 | 2.7 | 0.508 |
| Systolic blood pressure (mmHg) | 115.3 | 9.7 | 117.0 | 12.3 | 0.481 |
| Diastolic blood pressure (mmHg) | 69.5 | 7.7 | 72.1 | 6.7 | 0.105 |

Data presented as mean and standard deviation (SD). *Abbreviations*: ALP, alkaline phosphatase; APOA1, apolipoprotein A1; APOB, apolipoprotein B; BMI, body mass index; GGT, gamma-glutamyl transferase; GPT, glutamic pyruvic transaminase; HDL-C, high-density lipoprotein cholesterol; HOMA-IR, homeostatic model assessment of insulin resistance index; LDL-C, low-density lipoprotein cholesterol; VAT, visceral adipose tissue.

**Figure S1.** Associations between plasma succinate levels and heart rate parameters in young individuals stratifying by sex (n=85). Results are derived from multiple linear regression models adjusted for BMI. Upper plot legends display the standardized regression coefficient (β), the adjusted R²adj, and the P-value. 95% confidence intervals are represented as dashed lines above and below the linear regression lines. Coloured shaded depicts significant associations (P<0.05). *Abbreviations*: BMI, body mass index.
